# Multiple strains probiotics appear to be the most effective probiotics in the prevention of necrotizing enterocolitis and mortality: An updated meta-analysis

**DOI:** 10.1371/journal.pone.0171579

**Published:** 2017-02-09

**Authors:** Hung-Yang Chang, Jin-Hua Chen, Jui-Hsing Chang, Hung-Chih Lin, Chien-Yu Lin, Chun-Chih Peng

**Affiliations:** 1 Department of Pediatrics, MacKay Children’s Hospital, Taipei, Taiwan; 2 Department of Medicine, Mackay Medical College, New Taipei City, Taiwan; 3 Department of Medical Technology, Jen-Teh Junior College of Medicine, Nursing and Management, Miaoli, Taiwan; 4 School of Public Health and Biostatistics Center, Taipei Medical University, Taipei, Taiwan; 5 Department of Pediatrics, Children’s Hospital and School of Chinese Medicine, China Medical University, Taichung, Taiwan; 6 Department of Pediatrics, Hsinchu MacKay Memorial Hospital, Hsinchu city, Taiwan; Cardiff University, UNITED KINGDOM

## Abstract

**Background:**

Some oral probiotics have been shown to prevent necrotizing enterocolitis (NEC) and decrease mortality effectively in preterm very low birth weight (PVLBW) infants. However, it is unclear whether a single probiotic or a mixture of probiotics is most effective for the prevention of NEC.

**Objective:**

A meta-analysis was conducted by reviewing the most up to date literature to investigate whether multiple strains probiotics are more effective than a single strain in reducing NEC and death in PVLBW infants.

**Data sources:**

Relevant studies were identified by searches of the MEDLINE, EMBASE, and Cochrane CENTRAL databases, from 2001 to 2016.

**Data extraction and synthesis:**

The inclusion criteria were randomized controlled trials of any enteral probiotic supplementation that was initiated within the first 7 days and continued for at least 14 days in preterm infants (≤ 34 weeks’ gestation) and/or those of a birth weight ≤1500 g.

**Results:**

A total of 25 trials (*n* = 7345 infants) were eligible for inclusion in the meta-analysis using a fixed-effects model. Multiple strains probiotics were associated with a marked reduction in the incidence of NEC, with a pooled OR of 0.36 (95% CI, 0.24–0.53; *P* < .00001). Single strain probiotic using Lactobacillus species had a borderline effect in reducing NEC (OR of 0.60; 95% CI 0.36–1.0; *P* = .05), but not mortality. Multiple strains probiotics had a greater effectiveness in reducing mortality and were associated with a pooled OR of 0.58 (95% CI, 0.43–0.79; *P* = .0006). Trials using single strain of Bifidobacterium species and Saccharomyces boulardii did not reveal any beneficial effects in terms of reducing NEC or mortality.

**Conclusion:**

This updated report found that multiple strains probiotics appear to be the most feasible and effective strategy for the prevention of NEC and reduction of mortality in PVLBW neonates. Further clinical trials should focus on which probiotic combinations are most effective.

## Introduction

Necrotizing enterocolitis (NEC) remains the most common acquired gastrointestinal and surgical emergency in preterm very low birth weight (PVLBW) infants. The incidence of NEC ≥ stage 2 varies from 2.6% to 28.0% of PVLBW infants, with associated mortality ranging between 16% and 42% [1,2]. Preterm infants with NEC are at risk of long-term complications, including neurodevelopmental impairment, short bowel syndrome, and growth retardation [3,4]. Though there are significant morbidities associated with NEC, few safe and effective therapies are available to prevent this disastrous condition [5]. Available strategies for primary prevention of NEC include antenatal glucocorticoids, breast milk feeding, fluid restriction, and the use of probiotics [4,5]. However, during the past decade, only probiotics have been studied extensively in terms of the prevention of neonatal NEC in prospective randomized control trials (RCTs). Many meta-analyses of RCTs confirmed that oral probiotics effectively prevent NEC and death [6–14]. Although systemic reviews of single strain has been conducted [12,14], clinicians are facing challenges in assessing which probiotics are most effective in PVLBW infants. Unfortunately, published studies have used a variety of different single or multiple strains probiotic with different target populations. Relatively little is known about whether single strain probiotic alone or multiple strains are most effective in the prevention of NEC and death in PVLBW infants.

Recent articles have shown a link between NEC and a lack of microbiotal diversity [15–17]. One review article suggested that mixtures of probiotics were more beneficial than single strains for gut and immune function [18]. Our animal model also revealed that multiple strains probiotics were more effective in the prevention of NEC [19]. Furthermore, NEC does not usually occur after a gestation age of 34 weeks. We thus hypothesize that multiple strains probiotics are more effective in the prevention of NEC and death for preterm infants below a gestation age of 34 weeks. We conducted a meta-analysis by systematically reviewing the most up to date evidence available in the literature to investigate whether multiple strains probiotics are more effective than single-strain probiotics for reducing NEC and death in preterm infants.

## Materials and methods

### Search strategy and study selection

This meta-analysis was conducted according to PRISMA guidelines (http://www.prisma-statement.org/) (S1 PRISMA Checklist). Any trials following prespecified criteria were enrolled in the analysis: (a) RCTs involving PVLBW infants (≤ 34 weeks' gestation or birth weight ≤ 1500 g by mean or median) and reporting on NEC ≥ stage 2 by the Modified Bell staging criteria [20] and/or death, and (b) enteral administration of any probiotic commenced within the first 7 days of life and continued for at least 28 days. We searched the PubMed, Embase, and CBM databases for studies published from January 2001 to June 2016, with the terms “extremely low birth weight infant” or “very low birth weight infant” or “premature infant” or “preterm infant” and “Lactobacillus” or “probiotics” or “Saccharomyces” or “Bifidobacterium”. There was no language restriction. Similar studies and review articles reference lists in the references were also searched. The primary outcome was the efficacy of probiotic supplementation in preventing NEC ≥ stage 2. The secondary outcome was mortality before the infant was discharged.

### Data extraction

Two authors (H.Y.C. and H.C.L.) independently conducted the literature search. Information regarding study inclusion, study design, key characteristics, and outcomes was extracted independently by the 2 reviewers using a standardized data collection form. Inconsistencies were resolved by involving a third author (J.H.C.) or by discussion between all authors.

### Statistical analysis

To assess the between-study heterogeneity more precisely, both the *χ*^2^-based Q statistic test (Cochran Q statistic) to test for heterogeneity and the *I*^2^ statistic to quantify the proportion of the total variation attributable to heterogeneity were used. For each meta-analysis, the Cochran Q statistic was first calculated to assess the heterogeneity of the included trials. For *P* values less than .10, the assumption of homogeneity was deemed invalid. The outcome for our topic was binary in each trial; for example, whether NEC (or death) or not. The effects of probiotics for NEC (or death) were measured as odds ratios (ORs). For each trial, the OR is shown with a 95% confidence interval (95% CI). For NEC or mortality, a forest plot was used. For the meta-analysis, both the fixed-effects model and the random-effects model were considered. Homogeneity existed between almost all trials in our pooled study. The *I*^*2*^ of all trials or subgrouped trials was approximately zero. The fixed-effects model was considered to pool the estimators. Publication bias was investigated by funnel plot, and an asymmetric funnel plot suggested possible publication bias. Another way to explore the publication bias was the use of Egger’s regression test, which evaluated whether the intercept was significant. The significance level in the association test and the publication bias of our research were 0.05. Statistical analyses were performed using version 2 of the Comprehensive Meta-Analysis program (USA, 2006) and Review Manager (Cochrane Collaboration, Nordic Cochrane Centre) 5.1.

## Results

### Description of studies

**Fig 1:**
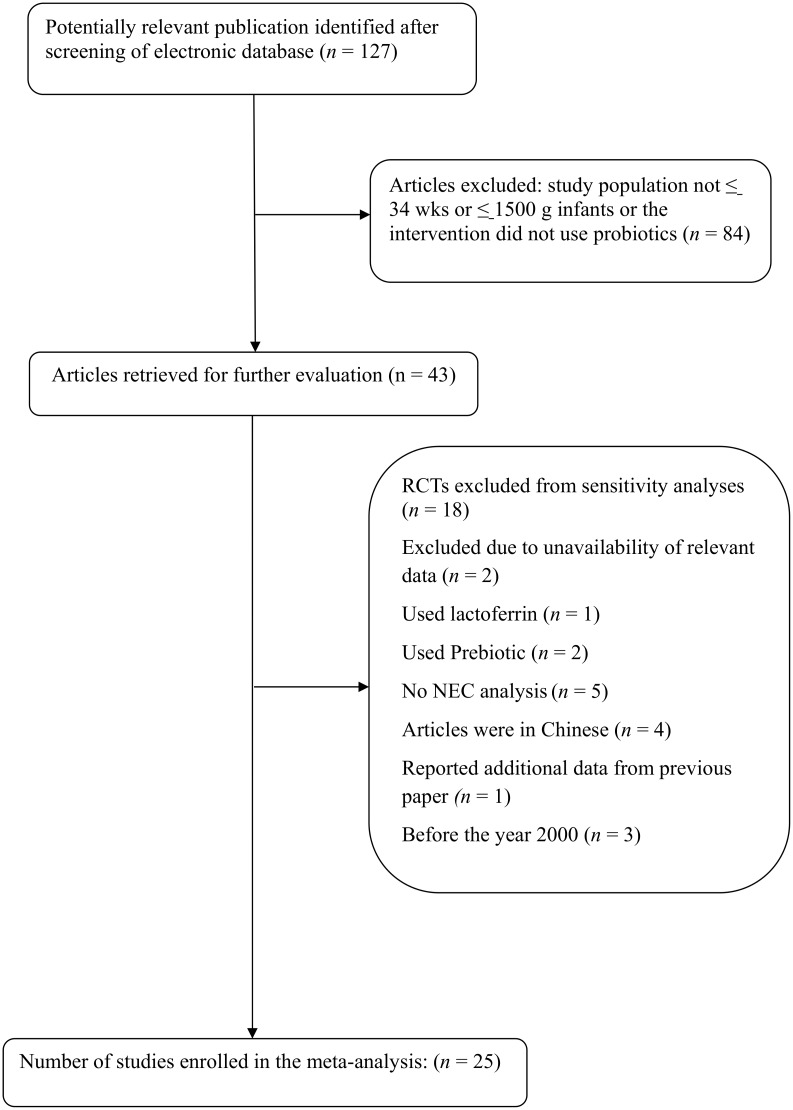
Flowchart showing the selection of studies for inclusion in the meta-analysis.

In total, there were 123 studies identified through electronic searches; 39 trials met the inclusion criteria and were selected to be read in full text (Fig 1). Eighteen studies were excluded for the following reasons: two studies had a lack of relevant data [21,22], one study used both lactoferrin and probiotics [23], two study used both prebiotics and probiotics [24,25], five studies did not assess the NEC outcomes [26–30], the full text could not be extracted in four studies [31–34], one study was a report of additional data from a previous paper [35], and three trials were conducted before 2000 [36–38]. After eliminating these trials, this review included data from 25 RCTs [39–63]. Two studies only enrolled infants with a birth weight under 1500 g [51,57]. A total of 7345 infants were included, 3679 in the probiotics group and 3666 in the control group. Each study was evaluated by Jadad score (Table 1). Of the 25 studies included in the analyses, fourteen studies used a single strain of probiotic (5 used a Lactobacillus strain [39,43,49,51,58], 6 used a Bifidobacterium strain [44,48,59,61–63], 3 used Saccharomyces boulardii [40,54,56]). Of the 5 Lactobacillus trials, 2 used Lactobacillus rhamnosus strain [39,43], 2 used Lactobacillus reuteri strain [51,58], and 1 used Lactobacillus sporogenes strain [49]. Of the 6 Bifidobacterium trials, 2 used Bifidobacterium lactis [44,48], 2 used Bifidobacterium breve [61,63], 1 used Bifidobacterium bifidus [59], and 1 used Bifidobacterium clausii [62]. Eleven studies used multiple strains probiotics, including 8 studies that utilized a combination of Bifidobacterium strain and Lactobacillus strain [42,45–47,50,52,57,60], and 3 studies that used a mixture of multiple probiotics strains [41,53,55].

**Table 1:** Characteristics of the trials included in the analysis.

Study	Participants		Gestation or birth weight	Probiotic agents	Outcomes	Jadad score
	Probiotics	Placebo				
Dani, 2002 [39]	295	290	< 33 wk, < 1500 g	L. rhamnosus GG	NEC	4
Costalos, 2003 [40]	51	36	28–32 wk	S. boulardii	NEC	5
Bin-Nun, 2005 [41]	72	73	< 1500 g	B. infantis, B. bifidus, Strepto. thermophiles	NEC, mortality	3
Lin, 2005 [42]	180	187	< 1500 g	L. acidophilus, B. bifidum	NEC, mortality	5
Manzoni, 2006 [43]	39	41	< 1500 g	L. rhamnosus GG	NEC, mortality	4
Stratiki, 2007 [44]	41	36	27–37wk	B. lactis	NEC	3
Lin, 2008 [45]	217	217	< 34 wk, < 1500 g	L. acidophilus, B. bifidum	NEC, mortality	5
Rouge, 2009 [46]	45	49	< 32 wk, < 1500 g	B. longum, L. rhamnosus GG	NEC, mortality	5
Samanta, 2009 [47]	91	95	< 32 wk, < 1500 g	B. infantis, B. bifidum, B. longum, L. acidophilus	NEC, mortality	3
Mihatsch, 2010 [48]	91	89	< 30 wk, < 1500 g	B. lactis	NEC, mortality	4
Sari, 2011 [49]	110	111	< 33 wk, < 1500 g	L. sporogenes	NEC, mortality	4
Braga, 2011 [50]	119	112	750–1499 g	L. casei, B. breve	NEC, mortality	5
Rojas, 2012 [51]	176	184	< 1500 g*	L. reuteri	NEC	5
Al-Hosni, 2012 [52]	50	51	501–1000g	L. rhamnosus GG, B. infantis	NEC, mortality	3
Fernandez-Carrocera, 2013 [53]	75	75	< 1500 g	L. acidophilus, L. rhamnosus, L. casei, L. plantarum, B. infantis, Strepto. thermophillus	NEC, mortality	5
Demirel, 2013 [54]	135	136	≤ 32 wk, ≤ 1500 g	S. boulardii	NEC, mortality	4
Jacobs, 2013 [55]	548	551	< 32 wk, < 1500 g	B. infantis, Strepto. thermophilus, B. lactis	NEC, mortality	4
Serce, 2013 [56]	104	104	≤ 32 wk, ≤ 1500g	S. boulardii	NEC, mortality	4
Roy, 2014 [57]	11	11	< 1000 g*	B. infantis, Lactobacillus acidophilus, B. lactis	NEC	4
Oncel, 2014 [58]	200	200	≤ 32 wk, ≤ 1500 g	L. reuteri	NEC, mortality	4
Totsu, 2014 [59]	153	130	< 1500 g	B. bifidus	NEC, mortality	4
Saengtawesin 2014 [60]	31	29	< 34 wk, < 1500 g	L. acidophilus, B. bifidum	NEC, mortality	3
Patole 2014 [61]	74	76	< 33 wk	B. breve M-16V	NEC, mortality	5
Tewari 2015 [62]	121	123	< 34 wk	B. clausii	NEC, mortality	4
Costeloe 2016 [63]	650	660	< 30 wk	B. breve BBG-001	NEC, mortality	5

L: Lactobacillus; B: Bifidobacterium; S: Saccharomyces; Strepto: Streptococcus; NEC, necrotizing enterocolitis.

*only patients with a birth weight < 1500 g were included in these studies.

### Efficacy of all probiotics on NEC

**Fig 2:**
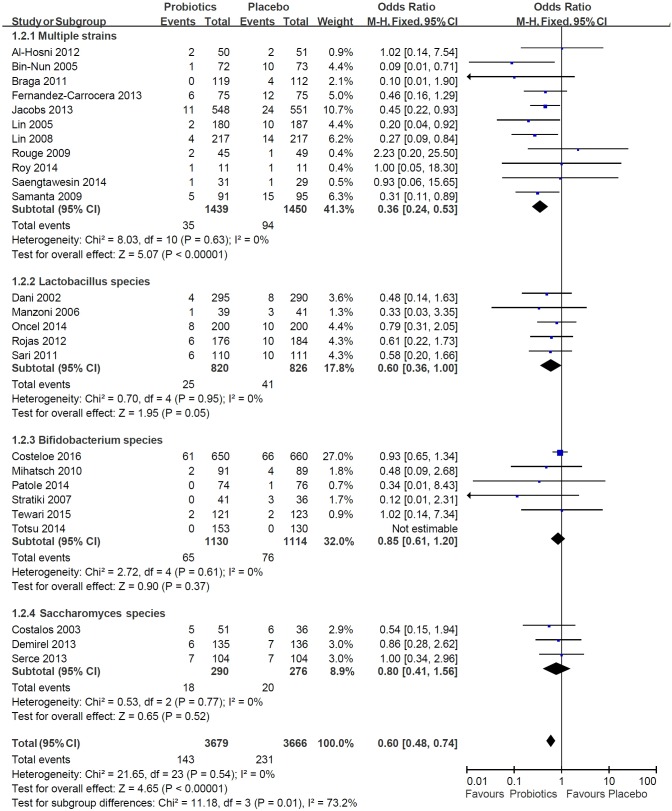
Meta-analysis of the efficacy of probiotics associated with the risk of NEC in preterm infants.

We combined the trials with all probiotics, such as multiple strains probiotics, Lactobacillus, Bifidobacterium, and Saccharomyces. The incidence of NEC stage ≥ 2 was 3.9% in the probiotics group, whereas it was 6.3% in the placebo group. The *P*-value of heterogeneity was 0.54, and *I*^*2*^ was 0%. The pooled OR was 0.60 with the fixed-effect model and the 95% CI was 0.48–0.74, *P* < .00001. The probiotics group had a lower risk of developing NEC than the placebo group (Fig 2).

#### Subgroup analysis: Efficacy of multiple strains probiotics versus single-strain probotics on NEC

In the multiple strains probiotics trials, the incidence of NEC stage ≥ 2 was 2.4% in the probiotics group, whereas in the placebo group it was 6.5%. In the Lactobacillus trials, the developed definite NEC stage ≥ 2 was 3.0% in the Lactobacillus group and 5.0% in the placebo group. In the Bifidobacterium trials, the developed definite NEC stage ≥ 2 was 5.8% in the Bifidobacterium group and 6.8% in the placebo group. In the Saccharomyces trials, the developed definite NEC stage ≥ 2 was 6.2% in the Saccharomyces group and 7.2% in the placebo group.

The meta-analysis showed that the placebo group had a higher risk of developing NEC than the multiple strains probiotics group (pooled OR: 0.36, 95% CI: 0.24–0.53, *P* < .00001, *I*^*2*^ = 0%). The odds of NEC occurring in the placebo group were higher than those in the single-strain probiotics groups, but these differences did not reach statistical significance (Fig 2). Single strain probiotic using Lactobacillus species had a borderline effect in reducing NEC (OR of 0.60; 95% CI 0.36–1.0; *P* = .05). Trials using single strain of Bifidobacterium species and Saccharomyces boulardii did not reveal any beneficial effects in terms of reducing NEC.

### Efficacy of probiotics on mortality

**Fig 3:**
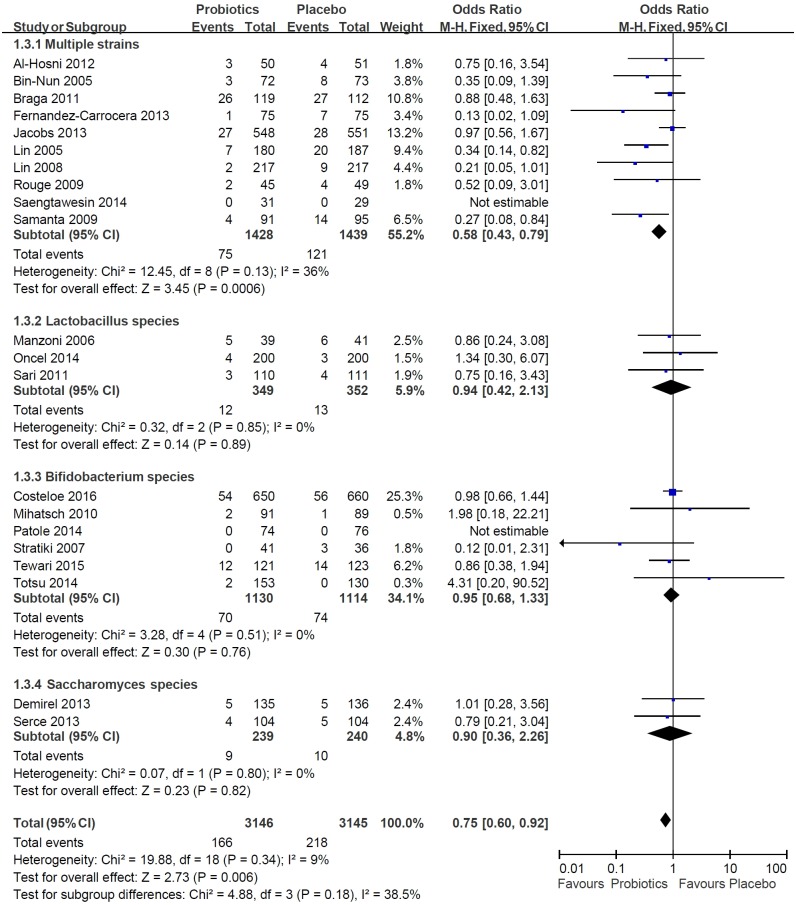
Meta-analysis of the efficacy of probiotics associated with the risk of death in preterm infants.

There were 21 trials that discussed the association between mortality and probiotics. The mortality rate in the probiotics group with all the tested probotics, including multiple strains probiotics, Lactobacillus, Bifidobacterium, and Saccharomyces, was 5.3%, whereas in the placebo group it was 6.9%. Probiotics reduced the risk of death by 25% relative to the placebo group (pooled OR: 0.75, 95% CI: 0.60–0.92, *P* = .006, *I*^*2*^ = 9%) (Fig 3).

#### Subgroup: Effect of combined probiotics and single-strain probiotics on mortality

We reported the effects of multiple strains probiotics, Lactobacillus. Bifidobacterium, and Saccharomyces on mortality separately. In the multiple strains probiotics cases, there were 5.3% and 8.4% mortality rates within the treatment and placebo groups, respectively.

Within the multiple strains probiotics group, the *P*-value of the heterogeneity test was 0.13, and *I*^*2*^ was 36%. The degree of heterogeneity was moderate. The pooled OR between multiple strains probiotics and death was 0.58, and the 95% CI was 0.43 to 0.79 (*P*-value = .0006). In the single-strain probiotics group, the pooled results in the probiotics group showed no statistical significance in relation to mortality as compared with the placebo group (Fig 3).

### Publication bias

A funnel plot was used to show the relationship between effect size and the standard error of estimator for each trial. A symmetric funnel plot would indicate that publication bias did not exist. Egger’s regression test, a weighted linear ordinary least squares regression, was used to explore the publication bias. The funnel plots of NEC and mortality looked generally symmetric separately. Egger’s tests for the intercepts were not statistically significant for NEC (*P*-value of intercept = .54) or mortality (*P*-value of intercept = .48). The Egger’s test corresponded with the symmetrical funnel plots. The funnel plots and Egger’s regression indicated no publication bias within our selected trials.

## Discussion

Our updated meta-analysis showed that multiple strains probiotics resulted in a marked reduction of the incidence of NEC and of the incidence of mortality in preterm infants ≤ 34 weeks' gestation or of a birth weight ≤ 1500 g.

NEC is a multifactorial disease, and its pathophysiology remains unclear. Several factors appear to contribute to the pathogenesis, including immaturity of multiple intestinal functions, such as gastrointestinal dysmotility, impaired digestive capacity, altered regulation of intestinal blood flow, barrier dysfunction, altered anti-inflammatory control, and impaired host defense. Frequent use of antibiotic therapy and anti-acid medications, followed by enteral feeding, are believed to increase the risk of NEC. Establishing a core microbiota of diverse commensal species is critical of PVLBW infants. Reasons for disruption or delay of this critical process include delivery modes, gestational age, birth weight, infectious diseases, antibiotics therapy, parenteral feeding, feeding type, and hospital period and environment. The dysbiosis of microbial succession [64,65], abnormal bacterial colonization, and lower bacterial diversity [15–17] in PVLBW infants also has been linked to the occurrence of NEC, which is the rationale for the need for probiotic supplements.

Although probiotics are the most promising treatment for reducing NEC, the use of different probiotics in all studies has made the selection of an optimal probiotic regimen difficult. Furthermore, preterm infants range up to ≤ 34 weeks’ gestational age, making it difficult to determine which preterm infants would benefit the most. The 25 trials that were enrolled in our review are summarized in Table 1 [39–53]. Our updated meta-analysis confirmed the results of other systematic reviews, which reported that probiotics prevent NEC and death. However, we focused on preterm infants of ≤ 34 weeks' gestation or of a birth weight ≤ 1500 g, who were a high-risk group for developing NEC or death, who had undergone enteral administration of probiotics commenced within the first 7 days of life and continued for at least 28 days. Our study discovered that multiple strains probiotics resulted in a marked reduction in NEC, which was comparable with results from one recent meta-analysis [12]. The potential mechanisms by which multiple strains probiotics may provide better protection from developing NEC might include increased diversity of the intestinal microbiota and offering healthy bacteria such as Lactobacillus and Bifidobacteria to balance normal microbiota in this vulnerable human population. However, it is unclear whether this is due to synergistic interactions between strains or a consequence of the higher probiotics doses.

Our updated meta-analysis further confirmed the results of other systematic reviews, which reported that probiotics supplements had a significant effect in reducing mortality in preterm infants of ≤ 34 weeks' gestation or of a birth weight ≤ 1500 g. However, after further analysis, 3 trials using a Lactobacillus strain alone, 6 trials using Bifidobacterium alone, and 2 trials using Saccharomyses alone did not reveal a beneficial effect on mortality. On the other hand, analyses from 10 studies showed a greater effectiveness of multiple strains probiotics in reducing mortality in PVLBW infants. It is unclear from this meta-analysis whether this was due to a reduction in NEC-related or sepsis-related deaths or other etiologies. In theory and animal studies, different probiotics may function differently in the modification of the intestinal immune system. Therefore, the effects of multiple strains probiotics probiotics on NEC or death may be acting through the synergetic effect, inhibiting the growth of pathogens, promoting up-regulation of the immune responses, or strengthening the mucosal barrier [17].

Concerns regarding safety issues and complications associated with the use of probiotics in these relatively immunocompromised preterm infants have been debated. However, the reported risk of sepsis due to translocation of the probiotics through the intestinal wall is extremely rare, and no apparent adverse effects were observed in any of the studies. Based on the currently available clinical trial results, probiotics use in preterm infants is generally considered to be safe. We did not analyze the efficacy of probiotics on sepsis, the time to full oral feeding, or the duration of hospitalization, because these items were not the primary or secondary outcomes of the enrolled studies. Only a fraction of the enrolled studies described these outcomes, which could not be analyzed because of publication bias.

There were several possible limitations that warranted careful review in this meta-analysis. First of all, the multiple strains probiotics treatment regimens varied widely. Questions regarding the optimal combination of species and dosing remain unanswered. Further studies directly comparing probiotic mixtures with single strains are warranted. Further research should also identify which multiple strains probiotics might be associated with improved health outcomes or enhance the preparation’s effectiveness. Second, publication bias may have existed for trials using single strain probiotic alone because of the limited study numbers. Probiotic effects are known to be strain specific, statistically and clinically significant benefits could relate to strain-specific differences. Although strainspecific meta-analysis data has been performed [12,14], recommendation cannot be made because of limited RCTs using the same single strain. Furthermore, there is significant heterogeneity among included studies. Some trials with small sample size and inadequate power, NEC or mortality as a secondary outcome are also affected our meta-analysis results. We should also point out that some of the probiotic products or placebos in the control group contained maltodextrin, which according to a recent animal study could increase the incidence of NEC [66]. However, our further analysis showed that probiotics containing maltodextrin had the same effect in terms of the prevention of NEC.

## Conclusions

Multiple strains probiotics, a therapeutic modification of the gut microbiota and restoring a healthy complement and diversity of commensal bacteria is the most logical approach to prevent NEC and death in PVLBW infants. The current evidence provided by this meta-analysis supported that multiple strains probiotics seemed to be the most feasible method and the most effective way to prevent NEC and reduce mortality in preterm infants of ≤ 34 weeks' gestation or of a birth weight ≤ 1500 g. Single strain probiotic using Lactobacillus species had a borderline effect in reducing NEC. Single strain of Bifidobacterium species and Saccharomyces boulardii did not reveal any beneficial effects in terms of reducing NEC or mortality. The optimal combination of species and dosing, long-term immune and neurodevelopment outcomes of probiotic supplementation in PVLBW infants still need to be explored in further studies.

## Supporting information

S1 PRISMA Checklist (DOC)
